# Current Estimate of Hearing Aid Utilization in the United States

**DOI:** 10.1097/ONO.0000000000000001

**Published:** 2021-08-23

**Authors:** Ashley M. Nassiri, Todd A. Ricketts, Matthew L. Carlson

**Affiliations:** 1 Department of Otolaryngology-Head and Neck Surgery, Mayo Clinic, Rochester, Minnesota; 2 Department of Hearing and Speech Sciences, Vanderbilt University Medical Center, Nashville, Tennessee; 3 Department of Neurologic Surgery, Mayo Clinic, Rochester, Minnesota.

**Keywords:** Hearing aid price, Hearing aids, Hearing amplification, Hearing loss, Prevalence

## Abstract

**Objective:**

To present key data from a private marketing report that characterizes US hearing aid (HA) utilization, HA candidate and user population sizes, and HA pricing.

**Patients:**

HA candidates and users in the United States.

**Interventions:**

Hearing amplification.

**Main Outcome Measures:**

HA utilization, HA candidate and user population sizes, HA market size and value, and HA pricing.

**Results:**

In 2015, an estimated 8.5 million HA users accounted for a total of 15.4 million individual HA devices in the United States. Approximately 81% of HA users owned bilateral devices. In 2015, approximately 87% of devices were purchased as replacements by current HA users, while the remaining 13% of devices were purchased by new HA users. HA utilization rates among the candidate population approximated 21% in 2015, which was stable over the 3-year study period. In 2015, there was a net increase of 696,060 individuals who met HA candidacy criteria but did not undergo treatment with HAs, adding to the backlog of 31.0 million untreated HA candidates who existed before that year. The HA market was valued at $6.0 billion in 2015, with an average retail selling price of $1798 per device ($3596 per pair). In the same year, the average manufacturer selling price was $495 per device ($990 per pair), or nearly 1 quarter of the retail price.

**Conclusions:**

HAs are substantially under-utilized in the United States with an annually growing backlog of untreated HA candidates.

Despite increasing recognition of the benefits of hearing aids (HAs) in treating hearing impairment and associated secondary sequelae, hearing loss persists as one of the most common, undertreated health conditions in the United States(1–6). Although it is understood that HAs are underused, recent utilization estimates are limited by outdated data(7–10), study samples not reflective of the diverse US population(7–10), or biases related to patient-reported data(4,11). While patient-facing surveys provide a wealth of knowledge around the patient experience of hearing loss and treatment in the United States, conclusions in regard to HA utilization are inherently limited by selection and recall biases (4,12). As a complement to data collected through patient surveys, a 2016 HA market report produced by iData Research Inc., a private market research firm, utilizes data from HA manufacturers, device sales, and diagnostic audiometry to estimate the number of HA candidates and users in the United States(13). The present report makes publicly available the most recent HA market report by iData Research Inc. in an effort to improve the understanding of HA utilization and market trends that may guide patient outreach, research efforts, and public policy.

## METHODS

### Description of Market Report

US HA utilization and market trends from the study period (2013–2015) were sourced from the US Market Report Suite for Hearing Devices published by iData Research Inc in 2016 (most recent available report) (13). While raw data and financial models are proprietary, models were informed by both objective data and expert opinion from HA device manufacturers and distributors. Data used for these estimates were pooled from the US Census, hospital and clinical practices (including Veteran Affairs estimates), academic publications, investor presentations, US Securities and Exchange Commission filings, and import and export databases. Hearing loss prevalence estimates were determined using US Census data. The authors approached iData Research to access the report; the iData Research team was not involved in the interpretation of the report, writing of this manuscript, or funding of this study.

### HA Candidacy Criteria and Pricing

The iData Research Inc. market report defined HA candidates as adults or children with unilateral or bilateral sensorineural hearing loss with a pure-tone average (PTA) between 25 and 90 dB (13,14). Individuals with a PTA greater than 90 dB and those with pure conductive hearing loss were considered cochlear implant and bone conduction device candidates, respectively, and were not included in the pool of HA candidates. HA device pricing is presented as an average selling price; manufacturer prices reflect the sales price paid to the manufacturer, and the retail price reflects the sales price paid to the retailer by the consumer (HA user). In some cases, prices may be inclusive of professional services including HA fitting, as these services may be bundled at certain institutions.

## RESULTS

In 2015, approximately 8.5 million HA users accounted for a total of 15.4 million individual HA devices in the United States (Table 1). Most individuals were binaural HA users; approximately 81% of HA users owned bilateral devices. During the 3-year study period, the number of annual new HA users and devices sold increased. Notably, an estimated 3.4 million individual HA devices were sold in 2015. Assuming 235,942 new HA users with a binaural rate of 81% in 2015, approximately 87% of devices sold in 2015 were purchased as replacements; in other words, only 13% of all HAs sold in 2015 were purchased by new users.

**TABLE 1.** Hearing aid market size from 2013 to 2015

Year	Total US population	Total US HA candidate population^*a*^	Total number of HA devices in use	Annual new HA used	Total HA users	Annual new HA users	Total untreated HA candidates
2013	317,083,319	38,684,165	14,552,983	-	8,084,990	-	30,599,175
2014	319,428,475	39,289,702	14,934,016	381,033	8,250,838	165,848	31,038,864
2015	321,773,631	40,221,704	15,389,360	455,344	8,486,780	235,942	31,734,924

^*a*^Includes adults and children with sensorineural hearing loss and pure-tone averages between 25 and 90 dB in at least 1 ear. Excludes patients with pure conductive hearing loss and profound sensorineural hearing loss. “-” indicates data not available.

HA indicates hearing aid.

The HA utilization rate, defined as the proportion of HA users (numerator) to the HA candidate population (denominator), was 21.1% in 2015, which was comparable to the utilization rate of 20.9% in 2013. Although both the HA user and candidate populations grew during the 3-year study period, the stable HA utilization rate can be attributed to proportional growth of both the HA user (numerator) and candidate (denominator) populations. Nevertheless, the HA candidate population grew by a greater absolute number of individuals compared with the HA user population resulting in a growing backlog of untreated HA candidates, which was estimated to be 31.7 million individuals in 2015.

The retail HA market demonstrated steady growth during the study period, with a market value of approximately $6.0 billion in 2015. In 2015, the average selling price of an individual HA device was $1798 in the retail market, and $495 in the manufacturer market; in other words, the retail price for each device was nearly 3.5 times greater than the manufacturer price. Table 2 summarizes the number of units sold and average selling price by HA type.

**TABLE 2.** Annual number of hearing aid devices sold and average selling price by device type in 2015

	Behind-the-ear	Receiver-in-canal	In-the-ear	In-the-canal	Completely-in-canal
Number of devices sold^*a*^	717,208	1,640,081	429,591	294,211	278,413
Average selling retail price^*a*^	$1690	$1875	$1671	$1725	$1894

^*a*^Numbers and prices reflect individual hearing aid device, not a pair.

## DISCUSSION

### Underutilization of HAs

Despite growing evidence that links hearing loss with cognitive impairment, dementia, depression, and social isolation, hearing loss remains one of the most prevalent, under-treated health conditions in the United States (5,15–18). In 2017, The Lancet Commission on Dementia Prevention, Intervention, and Care report estimated that up to 35% of dementia cases are potentially preventable, with hearing loss considered the largest modifiable risk factor (19). With growing evidence that suggests HAs may mitigate the psychosocial and cognitive effects of hearing impairment (6,20–26), efforts to reduce barriers to HA adoption have been suggested at the national level (27).

The present study reports a HA utilization rate of 21.1% in 2015, which is comparable to prior reports ranging from 10% to 34% (4,7–11). The iData Research Inc. market report suggests that HA utilization did not change substantially during the 3-year study period, which reflects proportional growth of both the HA user and candidate populations. In 2015, there was a net increase of 696,060 individuals who met HA candidacy criteria but did not undergo treatment with HAs, adding to the backlog of 31.0 million untreated HA candidates who existed before that year (Table 1). Altogether, these findings suggest that at present HA utilization rates, the backlog of untreated HA candidates will continue to grow.

The low utilization rate suggested by this and prior studies may chiefly be a result of barriers to initial HA adoption, rather than poor satisfaction with devices. Prior studies have demonstrated patient satisfaction and improvement of quality of life in HA users (4,28), a notion which is supported by the high proportion (87%) of annual replacement devices sold, suggesting continued use for many HA users. Instead, both diagnostic and treatment barriers to care may contribute to low HA utilization in the United States. Unlike other diseases where self-reported symptoms often correlate with diagnoses, self-reported hearing loss has not only been demonstrated to have low sensitivity in detecting true hearing impairment, but may also serve as a barrier to HA adoption in individuals with self-perceived normal hearing (29–31). While routine hearing screening programs exist for infants and children in the United States, hearing screenings in adults are not commonplace, which may contribute to underdiagnosis and therefore undertreatment of individuals with hearing impairment (32,33).

For patients diagnosed with hearing loss, low HA utilization is likely multifactorial and varies significantly according to socioeconomic status, race, education level, age, and severity of hearing loss (33–36). Individuals with mild or unilateral hearing loss are less likely to use HAs, which may be associated with self-perceived normal hearing (29,37,38). Not surprisingly, device cost persists as a major barrier to HA adoption as government and private insurers (with the exception of Veterans Health Administration) rarely offer HA coverage, with 77% of hearing-impaired Americans unable to afford HAs ($3596 per pair in 2015) (13,39,40). Nevertheless, HA utilization remains low even in health systems that fully cover the cost of the devices, suggesting that cost does not act alone as a barrier to HA adoption (41).

### Limitations

While inclusion criteria used in this marketing report are available, the models and raw data used to derive the figures presented in the iData Research Inc. marketing report are proprietary. The estimates of the HA candidate population, however, do align with prior studies that evaluated hearing loss prevalence within the US population (42,43). Importantly, this study uses population-based audiometric data to estimate the HA candidate population, which mitigates some of the potential inaccuracies associated with self-reported hearing loss and selection bias (29).

Efforts to characterize HA utilization are significantly impacted by the hearing device candidacy criteria used to determine the size and composition of the HA candidate population. As hearing device technology improves and device candidacy expands, patients with similar levels of hearing loss may have multiple treatment options. In an attempt to reduce overlaps in device candidacy, the iData Research Inc. market report defined HA candidates as individuals with unilateral or bilateral mild-to-severe sensorineural hearing loss, but excluded those with profound sensorineural hearing loss and pure conductive hearing loss. While this may reduce the overlap with cochlear implant and bone conduction device candidates, there exist patients with profound sensorineural hearing loss and conductive hearing loss who currently use HAs, which would effectively overestimate the HA user population and underestimate the HA candidate population. Furthermore, as cochlear implant indications have expanded, some patients with severe hearing loss may be cochlear implant candidates and users, as opposed to HA users, which effectively overestimates the HA candidate population. Although caveats in hearing device use and candidacy may impact cross-sectional utilization rates, the stagnant HA utilization rate over time draws attention to a compelling trend that reflects the overall lack of progress in improving HA penetration within the candidate population in the United States.

## CONCLUSION

HAs continue to be considerably under-utilized in the United States, as only 21% of HA candidates used HAs in 2015. Despite high rates of replacement purchases suggesting perceived HA user benefit, HA utilization did not increase over the 3-year study period. An improved understanding of HA utilization in the United States, and specifically across subpopulations, is required to guide clinical practice and outreach in an effort to improve diagnostics and treatment in hearing loss.

## FUNDING SOURCES

None declared.

## CONFLICT OF INTEREST

A.M.N. holds the position of Associate Editor for *Otology & Neurotology Open* and has been recused from reviewing or making editorial decisions for this manuscript; research funding from Cochlear Americas (unrelated to this study). M.L.C. received research funding from Cochlear Americas (unrelated to this study). T.A.R. discloses no conflicts of interest.

## DATA AVAILABILITY

The market report data used for this study is available for purchase through iData Research Inc.
